# A novel blood-free analytical framework for the quantification of neuroinflammatory load from TSPO PET Imaging

**DOI:** 10.21203/rs.3.rs-5924801/v1

**Published:** 2025-02-03

**Authors:** Lucia Maccioni, Ludovica Brusaferri, Leonardo Barzon, Julia J. Schubert, Maria A. Nettis, Oliver Cousins, Ivana Rosenzweig, Yuya Mizuno, Marta Vicente-Rodríguez, Nisha Singh, Tiago Reis Marques, Neil A. Harrison, Tim Fryer, Edward T. Bullmore, Diana Cash, Valeria Mondelli, Carmine Pariante, Oliver Howes, Federico E. Turkheimer, Marco L. Loggia, Mattia Veronese

**Affiliations:** 1 Department of Information Engineering, University of Padova, Padova, Italy; 2 Athinoula A. Martinos Center for Biomedical Imaging, Department of Radiology, Massachusetts General Hospital, Harvard Medical School, Charlestown, MA, USA; 3 Computer Science and Informatics, School of Engineering, London South Bank University, London, UK; 4 Institute of Psychology, Psychiatry and Neuroscience, King’s College London, London, UK; 5 South London and Maudsley NHS Foundation Trust, London, UK; 6 Department of Neuropsychiatry, Keio University School of Medicine, Tokyo, Japan.; 7 Departamento de Ciencias Farmacéuticas y de la Salud, Facultad de Farmacia, Universidad San Pablo-CEU, CEU Universities, Madrid, Spain; 8 Psychiatric Imaging Group, MRC London Institute of Medical Sciences (LMS), Hammersmith Hospital, Imperial College London, London, UK; 9 Cardiff University Brain Research Imaging Centre (CUBRIC), Cardiff University, Cardiff, UK; 10 Department of Clinical Neurosciences, School of Clinical Medicine, University of Cambridge, Cambridge, UK; 11 Department of Psychiatry, School of Clinical Medicine, University of Cambridge, Cambridge, UK; 12 Institute of Clinical Sciences (ICS), Faculty of Medicine, Imperial College London, Du Cane Road, London W12 0NN, UK; 13 Department of Anesthesia, Critical Care and Pain Medicine, Massachusetts General Hospital and Harvard Medical School, Charlestown, MA, USA

**Keywords:** blood-free quantification, neuroinflammation, normative modeling, PET, TSPO

## Abstract

Positron Emission Tomography (PET) of the 18 kDa translocator protein (TSPO) is critical for neuroinflammation studies but faces substantial methodological challenges. These include issues with arterial blood sampling for kinetic modeling, the absence of suitable reference regions, genetic polymorphisms affecting tracer affinity, altered blood-to-brain tracer delivery in inflammatory conditions, and high signal variability. This study presents a novel blood-free reference-free method for TSPO PET quantification, leveraging a logistic regression model to estimate the probability of TSPO overexpression across brain regions. Validation was performed on 323 human brain scans from five datasets and three radiotracers. The quantified TSPO topology in healthy controls showed strong concordance with the constitutive TSPO gene expression for all tracers. When using [^11^C]PBR28 PET data, the method replicated previous findings in schizophrenia, Alzheimer’s disease, chronic pain, and XBD173 blocking. However, model extension to [^18^F]DPA-714 and [^11^C]-(R)-PK11195 revealed small effect sizes and high variability, suggesting the need for tracer-specific model optimization. Finally, validation in a rat model of lipopolysaccharide-induced neuroinflammation confirmed previous evidence of increased brain TSPO uptake after a systemic challenge.

This novel non-invasive method provides individualized TSPO PET quantification, demonstrating broad applicability across TSPO PET tracers and imaging sites, assuming sufficient training data for model development.

## 1. Introduction

“Neuroinflammation” is a broad term ^1^ commonly adopted to describe a series of cellular and molecular responses enacted by the nervous system when it is facing a challenge to its homeostasis (e.g., cellular damage, bacterial/viral infection, etc.) ^2,3^. This complex set of processes that aim to restore the disturbed homeostasis includes phenotypic changes and/or changes in cell density, typically driven by microglia, astrocytes, and/or peripheral immune cells invading the central nervous system, and the release of cytokines/chemokines ^4–6^. While neuroinflammatory responses are often transient, certain abnormal conditions can prevent the restoration of normal function, and prolonged and chronic neuroimmune activation can turn this otherwise protective response into a pathological condition ^3,7^. Substantial evidence accumulated over the past decades indicates that neuroinflammation plays a critical role in the onset and progression of various brain disorders, including neuropsychiatric disorders, neurodegenerative diseases, brain injuries, glioma, stroke, and chronic pain conditions ^8–14^.

The 18 kDa translocator protein (TSPO) Positron Emission Tomography (PET) imaging represents the most widely used tool for in vivo quantification of neuroinflammation despite its lack of cellular specificity ^5,15–18^. Clinical interest in TSPO arises from the fact that, while minimally expressed in the healthy brain ^15^, TSPO expression notably increases in activated microglia, astrocytes, macrophages, and neurons during neuroinflammatory processes ^13,19–21^. Consequently, TSPO is often used as a biomarker of neuroinflammation across a wide spectrum of neurodegenerative, psychiatric, and inflammatory disorders ^22^.

However, quantification for this class of radioligand remains challenging ^23^. Standard blood-based kinetic modeling has practical and methodological problems ^24,25^, including the need for invasive catheterization for arterial blood sampling, the challenges of accurately measuring radioactivity in blood and plasma, and the need to correct for the radioactivity of tracer metabolites as well as for the plasma protein binding. The latter is particularly challenging, as the magnitude of TSPO radiotracers free plasma fraction is generally modest (<5%) ^22,23,26^ and sensitive to the presence of inflammatory proteins ^27–29^. An alternative to blood-based modeling is the use of a reference region for quantification ^30,31^. Such a region should ideally possess perfusion properties similar to the tissues of interest but exhibit minimal or no expression of the ligand’s target. While TSPO expression in the brain is relatively low, it is ubiquitously expressed across most cell types ^32^, which significantly complicates reference-based quantification. Additionally, the pros and cons of these approaches need to be carefully evaluated since the normalization for the signal in the reference region might limit, in some cases, the ability to detect the brain-wise effects of a disease ^33^.

The large and often unpredictable intra- and inter-individual biological variability in the TSPO PET signal adds to this complexity. This variability hampers the statistical power to detect group effects linked to specific disorders and may have contributed to inconsistent evidence regarding the role of neuroinflammatory processes in both physiological conditions and various brain pathologies ^3^. One possible explanation for this variability might be related to the unclear relationship between peripheral and central inflammation, as well as the role of blood-brain-barrier (BBB) alterations, which affect the rate of tracer delivery from blood to parenchyma and thus the measured activity of the tracer in the tissue ^29,34–36^. This aspect further complicates the quantification of TSPO brain density from PET imaging. Another source of variability arises from human genetic differences in tracer affinity ^37^, which affect the utility of the newer-generation TSPO radiotracers developed to address the limitations of [^11^C]-(*R*)-PK11195, such as its high non-specific binding and low target affinity ^22,23,38^. As a result of the single nucleotide polymorphism in the TSPO gene (rs6971), determining the substitution of the Ala147Thr amino-acid and thus variations in tracer binding affinity to the TSPO protein, individuals are classified into three different binding affinity classes: high-affinity binders (HAB), mixed-affinity binders (MAB), and low-affinity binders (LAB). Genotype analysis allows for the stratification of subjects into more homogeneous cohorts, but for several tracers LABs must be excluded from the study for the impossibility of providing a reliable signal (although third-generation radioligands like ER176 have sufficient sensitivity to image LABs). Other factors such as subjects’ sex, age, and body mass index (BMI) have been shown to affect the baseline brain inflammatory load and, consequently, TSPO binding ^38–40^. Taken together, this evidence underscores the importance of understanding the physiological mechanisms underlying TSPO PET variability. Such insights are crucial for developing statistical models that can effectively account for these biological sources of variability in the PET signal, thereby enabling more accurate quantification of individual neuroinflammatory status.

In this work, we propose a new non-invasive blood-free reference-free analytical framework for the quantification of dynamic TSPO PET imaging based on a logistic regression model. Logistic regression is a statistical method, generally adopted for binary classification problems, which allows the prediction of the probability of one possible outcome to occur. The use of a logistic function transforms the linear combination of a set of independent variables (i.e. the model covariates or predictors) into a probability value between 0 and 1. Here, we defined a logistic regression model that takes brain TSPO PET raw time activity curves (TACs) as predictors to provide a probability measure of the tissue to manifest an overexpression of TSPO. The model is enriched with additional inputs including the estimate of tracer perfusion and tracer blood-to-brain extraction and a set of individual covariates representing possible confounders of interest for the brain TSPO signal.

Our approach was tested using historical TSPO databases from two academic institutions (i.e. the *Centre for Neuroimaging Sciences at King’s College London (KCL)* and the *Athinoula A. Martinos Center of Biomedical Imaging, Massachusetts General Hospital*) on a total of 323 human brain PET scans, from 5 different PET scanners, and utilizing three TSPO tracers (i.e. [^11^C]PBR28, [^18^F]DPA-714 and [^11^C]-(*R*)-PK11195). Firstly, the model was defined, optimized, and tested on [^11^C]PBR28 data from healthy controls and patients with CNS disorders. Specifically, the physiological topology of the brain TSPO density was investigated in healthy conditions; then, the ability of the model to unveil alterations in regional neuroinflammatory load was tested for different published studies on patients with psychiatric disorders (risk of psychosis and schizophrenia ^41^), neurodegenerative disease (Alzheimer’s disease ^42^), and a chronic pain condition (fibromyalgia ^43^), as well as on a target blocking ^44^ and a test-retest study ^42^. Then, the possibility of generalizing the model to different TSPO tracers was evaluated on [^18^F]DPA-714 and [^11^C]-(*R*)-PK11195 PET scans on healthy volunteers, individuals with mild cognitive impairment ^45^, and depressive disorder patients ^46^. Finally, the ability of the logistic model to unveil the TSPO PET signal increase linked to brain inflammation was tested on a rat model of lipopolysaccharide (LPS) induced neuroinflammation.

## 2. Material and Methods

### 2.1. Logistic regression model

Our novel approach for TSPO PET quantification exploits the theory of logistic regression models ^47^. These regression approaches allow the prediction of the probability 𝑝 that a given input belongs to a particular class based on a set of predictors or covariates describing that specific input. This is done by adopting a logistic function that transforms the linear combination of the input variables into a probability p ranging between 0 and 1. The mathematical formulation of the model is reported in Eq.1, where $X_{j}$ and $\beta_{j}$ represents respectively the j-th input covariate and its corresponding coefficient, $\beta_{0}$ represents model intercept, n is the number of input covariates, and ln represents the natural logarithm function.

(Eq.1) $$ln\left(\frac{p}{1-p}\right)=\beta_{0}+\sum_{j=1}^{n}\beta_{j}X_{j}$$

Upon maximum likelihood estimation of the coefficients $\beta_{j}$ on a training set, the model can be applied to unseen data. This approach is generally adopted in classification tasks: upon selection of a threshold for the probability p of the input belonging to a specific class ($p_{TH}$), we assign the input to the class (outcome 1) if $p>\;p_{TH}$, otherwise to the complementary class (outcome 0).

Here we adapted the logistic model for brain TSPO PET imaging, to distinguish between regions with normal TSPO expression (outcome 0) from regions with TSPO overexpression (outcome 1). The probability distribution of the latter is hence used as a proxy of neuroinflammatory load. The model relies on the following assumptions:

1. The higher the PET signal measured by the scanner in a given volume of the brain, the higher the concentration of radiotracer in that volume.
2. The overall concentration of the tracer in a given volume of brain is the sum of many components which include the inflammatory status of brain parenchyma, the tracer perfusion, and extraction through the brain barriers.
3. The explicit modeling of TSPO tracer kinetics through individual covariates allows us to explain the variability of constitutive TSPO signal, and to distinguish between normative conditions and altered states.
4. The effect of each covariate is equivalent for all the regions across the brain and cerebellum cortex (model coefficients are fixed for all regions).

**Figure 1.**
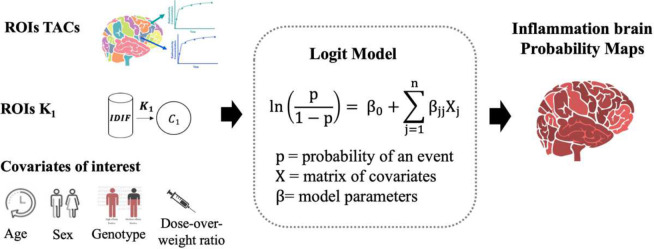
Logistic regression model for TSPO PET quantification The figure reports a schematic representation of the logistic regression model, with the inputs and outputs of the model. The method takes as input ROIs TSPO TACs, ROIs *K*_*1*_, computed with a novel noninvasive methodology adopting an IDIF, and a set of individual covariates and gives as output ROIs **p**_**TSPO**_. [ROI=region of interest; *K*_*1*_=blood-to-brain delivery rate; IDIF=image-derived input function; **p**_**TSPO**_= ROI probability of manifesting an overexpression of TSPO]

These assumptions translate into a logistic model that takes as predictors the brain regional TSPO TAC and provides the probability of a specific region manifesting an overexpression of TSPO (**p**_**TSPO**_). Additional predictors, representing possible confounders for the brain TSPO signal, included a measure of the regional tracer blood-to-brain delivery rate provided by the kinetic parameter *K*_*1*_, computed with a novel noninvasive methodology adopting an image-derived input function (IDIF) ^48^, and a set of individual covariates including age, sex, TSPO genotype (HAB and MAB), and the injected dose normalized for patient weight. A schematic representation of the logistic regression model can be found in Figure 1.

Operationally, the proposed methodological framework involves two main steps. Firstly, the logistic regression model is trained on a population of HCs for the classification of regions of interest (ROIs) with known TSPO expressions, organized into two separate classes with constitutive low and high TSPO expression and representing non-inflamed and inflamed tissue, respectively. Once the model coefficients are defined, the logistic regression can be applied to unseen data (either new PET scans of independent subjects or brain region TACs not included in the model), where for a given TAC, it returns **p**_**TSPO**_ which is used as the main parameter of interest.

Conceptually, this logistic regression approach is similar to the supervised clustering approach already used in TSPO PET studies ^31^. Both approaches use a pre-defined set of classes with known TSPO density as a proxy of normal and inflamed brain tissues. However, while with supervised clustering the tissue classification focuses on identifying a tissue with low TSPO density to be used as a reference region for tissue modeling, here the classification turns into an indirect quantification of TSPO density.

### 2.2. Study participants and datasets

A total of 323 TSPO PET scans were included in this study. Data included 5 datasets of [^11^C]PBR28, [^18^F]DPA-714, and [^11^C]-(*R*)-PK11195 dynamic PET scans gathered from *King’s College London (KCL) and Athinoula A. Martinos Center for Biomedical Imaging, Massachusetts General Hospital (MGH)* historical databases. Information on datasets is summarized in Table 1.

Each study was approved by local ethics committees and institutional review boards before starting, including the *Queen Square London Ethical Committee*, *South Central Berkshire NRES Committee*, *Hammersmith Research Ethics Committee*, *London-Bloomsbury ethics committee*, *National Research Ethics Service Committee East of England Cambridge Central*, and the *UK Administration of Radioactive Substances Advisory Committee* for *KCL* data, the institutional review board, the *Radioactive Drug Research Committee*, the *Food and Drug administration* and the *Partners Human Research Committee* for *MGH* data. All the studies were conducted in accordance with the *Declaration of Helsinki* and all participants provided informed consent to participate. Despite differences across imaging sites in scanner types, data acquisition, and PET reconstruction parameters, all protocols included a continuous dynamic acquisition following a bolus injection of the tracer. PET data were corrected for random and scattered coincidences and tissue attenuation. Given the genetic rs6971 polymorphism of the TSPO gene ^37,49^, all participants were genotyped before scanning, and no LABs were included in the original studies. Structural T1-weighted (T1w) Magnetic Resonance (MR) images were also acquired for each participant and used for image processing and atlas-based anatomical brain segmentation.

#### Dataset 1. KCL [^11^C]PBR28 scans

A total of 118 dynamic [^11^C]PBR28 PET and MR images collected both from healthy volunteers and patients were gathered from the KCL historical database (Age: 38±19 years; Sex: 84 male and 34 female; Genotype: 87 HABs and 31 MABs).

The dataset included 72 healthy controls (HC) with no family history of psychiatric or neurological conditions ^28,41,50^, 14 ultra-high-risk of psychosis (UHR) subjects (Bloomfield et al., 2016), 14 schizophrenia (SCZ) patients (Bloomfield et al., 2016), and 5 Alzheimer’s disease (AD) patients ^42^ having test-retest scans with a mean inter-scan time of 82 ± 10.2 days, starting at the same time of the day, and with no significant difference between injected doses (test = 339.0 ± 4.6 MBq, retest = 350.8 ± 14.2 MBq, p = 0.154)). In addition, 7 subjects with schizophrenia (6 of which were included among the 14 SCZ patients) had 2 scans, before and immediately after receiving a 90mg oral dose of the TSPO antagonist XBD173, designed to reach between 66% and 77% of TSPO brain occupancy (XDB173 blocking before and XDB173 blocking after). The second scan was acquired approximately two years after the first acquisition - except for one subject for which the second scan was acquired only 1 week after - at a similar time of the day to the first one ^44^;

More details on participants and data acquisition can be found in the original references ^28,41,42,44,50^. However, all acquisition protocols included an initial low-dose head computer tomography (CT) scan, acquired for attenuation and scatter correction, using a *Siemens Biograph^™^ TruePoint^™^ PET·CT scanner* (*Siemens Medical Systems, Germany*) and a dynamic PET scan after a bolus injection of [^11^C]PBR28 (Injected Dose: 328.10 ± 32.79 MBq). The radiopharmaceutical preparation protocol was consistent in all the studies and was performed on-site immediately prior to use according to local guidelines and regulations.

For all the data, dynamic PET acquisition lasted 90 min and was binned into 26 frames (durations: 8×15s, 3×1min, 5×2min, 5×5min, 5×10min), except for the 10 AD dynamic image data which were collected over 60 min and binned into 23 frames (durations: 8×15s, 3×1min, 5×2min, 5×5min, 2×10min). PET images were reconstructed using filtered back projection, with a 5mm isotropic Gaussian smoothing, and corrected for random coincidences, attenuation, and scatter effects. T1w MR brain scan data were collected using a *Siemens 3T MR* scanner (either a *Siemens Tim Trio* or *Siemens MAGNETOM Verio*). In the case of AD patients, a clinical 1.5T T1w MR was carried out instead.

#### Dataset 2. MGH [^11^C]PBR28 scans (scanner 1)

Data included a total of 46 [^11^C]PBR28 scans acquired from 27 HCs and 19 patients affected by fibromyalgia (FM) (Age: 47 ± 13 years; Sex: 15 male and 31 female; Genotype: 30 HABs and 16 MABs). Data were gathered from previous studies ^43,51,52^. In all studies, dynamic PET data acquisition was performed for 90 min after bolus injection of [^11^C]PBR28 tracer (Injected Dose: 488.16 ± 54.99 MBq) with an integrated PET/MR scanner consisting of a dedicated brain avalanche photodiode-based PET scanner (Kolb et al., 2012) in the bore of a Siemens 3T Tim Trio MRI. Data were reconstructed using the 3D ordinary Poisson ordered subset expectation maximization (OP-OSEM) algorithm provided by the manufacturer and binned into 28 frames (duration: 8×10s, 3×20s, 2×30s, 1×1min, 1×2min, 1×3min, 8×5min, 4×10min). A multi-echo MPRAGE volume was acquired before tracer injection for anatomical localization and generation of attenuation correction maps using an in-house developed MR-based approach ^53^. [^11^C]PBR28 was produced in-house using a procedure modified from the literature ^54^.

#### Dataset 3. MGH [^11^C]PBR28 scans (scanner 2)

Data included dynamic [^11^C]PBR28 PET/MR images from 26 HCs (Age: 55 ± 15 years; Sex: 12 male and 14 female; Genotype: 13 HABs and 13 MABs) simultaneously collected on a *Siemens Biograph mMR* whole-body PET/MR scanner. Full details on the acquisition can be found in (Morrissey et al., 2023). Dynamic PET data were acquired for 90 minutes after a bolus injection of [^11^C]PBR28 (Injected Dose: 519.44 ± 63.04 MBq), binned into 28 frames (duration: 8×10s, 3×20s, 2×30s, 1×1min, 1×2min, 1×3min, 8×5min, 4×10min) and reconstructed using the Ordered Subset Expectation Maximisation (OSEM) with four iterations, 21 subsets, and a 3 mm Full Width Half Maximum (FWHM) Gaussian smoothing. As for dataset 2, [^11^C]PBR28 tracer was produced in-house, and structural T1w images were used for the generation of attenuation correction maps ^53^.

#### Dataset 4. KCL [^18^F]DPA-714 scans

Data included 57 [^18^F]DPA-714 scans performed on 41 HCs and 16 subjects at increased risk of Alzheimer’s disease (Age: 39 ± 19 years; Sex: 31 male and 26 female; Genotype: 37 HABs and 20 MABs). Specifically, the 16 patients at risk included 8 individuals who were carriers of the rare p.R47H genetic variant of the triggering receptor expressed on myeloid cells 2 immune receptor (TREM2) and 8 non-carriers with mild cognitive impairment (MCI) ^45^. Dynamic data were collected on a SIEMENS Biograph mMR PET/MRI over 60 min after bolus injection of [^18^F]DPA-714 (Injected Dose: 186.88 ± 10.00 MBq) and binned into 26 frames (1×1min, 8×15s, 3×1min, 5×2min, 9×5min). All participants underwent a CT head scan which was used for attenuation correction ^57^, and a T1w MRI on the *3T SIEMENS Biograph mMR*.

#### Dataset 5. KCL [^11^C]-(*R*)-PK11195 scans

The dataset included 76 [^11^C]-(*R*)-PK11195 PET/MR scans collected from 51 patients with mild-moderate depression (MD) and 25 age-matched HCs (Age: 37 ± 8 years; Sex: 26 male and 50 female) recruited as part of the BIODEP (Biomarkers in Depression) study (NIMA consortium; https://www.neuroimmunology.org.uk/biodep/). Details on inclusion criteria and data acquisition can be found in ^46^.

Dynamic PET data were acquired for 60 minutes after [^11^C]-(*R*)-PK11195 injection (Injected Dose: 365.53 ± 50.89 MBq) on a *GE SIGNA* PET/MR (GE Healthcare, Waukesha, WI). Dynamic data were corrected for dead time, randoms, scatter, and decay directly on the scanner. Attenuation correction was performed with a multisubject atlas method ^58,59^ and improvements for the MRI brain coil component ^60^. Dynamic sinograms were reconstructed using time-of-flight ordered subsets expectation maximization, with 6 iterations, 16 subsets, and no smoothing, and binned into 17 frames (duration: 4×15s, 4×1min, 7×5min, 2×10min). All subjects underwent a high-resolution T1w brain MRI during PET acquisition.

### 2.3. Image preprocessing

Data were analyzed using different combinations of in-house codes and neuroimaging analysis software including *Statistical Parametric Mapping 8* (http://www.fil.ion.ucl.ac.uk/spm), *FSL* (http://www.fsl.fmrib.ox.ac.uk/fsl) and *MIAKAT*^™^ (http://www.miakat.org/MIAKAT2/index.html).

All pipelines included a step of motion correction of the dynamic PET data, the computation of integral PET images, the derivation of brain tissue masks from structural MR images (performed using the *FreeSurfer* package), and the registration of brain and tissue masks, as well as of a neuroanatomical atlas – the *CIC* atlas version 2.0 ^61^ - to the subject’s native PET space for the definition of ROIs. The CIC atlas was masked using a grey matter mask derived from the *FreeSurfer* parcellation image to exclude voxels belonging to white matter, cerebrospinal fluid, or outside the brain and then used for the computation of ROIs mean regional TACs for each subject. 116 out of 125 available ROIs from the CIC atlas, defining cortical, subcortical, and cerebellum regions, were retained for subsequent analyses.

Following the approach described in ^48^, an image-derived input function (IDIF) was extracted from the subjects’ dynamic PET images via segmentation of blood voxels in the carotid siphons by intensity thresholding, and selection of the voxels with higher between dynamics correlation; mean blood TAC was thus computed and fitted with a tri-exponential model.

Regional tracer delivery rate (kinetic microparameter *K*_*1*_) was finally computed for each ROI by application of a recently developed non-invasive and simplified methodologic framework consisting of fitting the first minutes (within 10 minutes) of the tracer kinetic in the tissue with a simplified compartmental model with 1 single irreversible compartment and IDIF. Further details on methodology and procedures for IDIF and *K*_*1*_ estimation can be found in ^48^. The choice of optimal fitting time window – allowing the satisfaction of model hypotheses - was based on dynamic data time resolution and the specific characteristics of tracer kinetics and metabolism. A four-minute window was adopted for [^11^C]PBR28, while a five-minute window for the [^18^F]DPA-714 and [^11^C]-(*R*)-PK11195 scans.

### 2.4. Validation of the logistic regression with [^11^C]PBR28 PET imaging

#### Model training and application in healthy controls

A TSPO expression map was derived from mRNA data of 6 post-mortem brains from the *Allen Human Brain Atlas* (https://human.brain-map.org/microarray/search/show?exact_match=false&search_term=%22TSPO%22&search_type=gene) with the *Abagen* toolbox using the *CIC* volumetric atlas in MNI space. TSPO expression map allowed for the investigation of the topological pattern of TSPO expression and the identification of a subset of ROIs with the lowest (*low-expression ROIs*) and highest (*high-expression ROIs*) TSPO expression to be adopted for model training.

The training was performed on a dataset of 72 [^11^C]PBR28 PET scans of HCs (Dataset 1). The model regressors included:

- Regional TACs time samples; each ROI TAC was normalized to standardized uptake value (SUV) to correct for differences in dose-over-weight ratio and then interpolated on a sparser time grid ([1.25 4.5 13.5 30 50 75] min) to reduce collinearity between variables
- Regional *K*_*1*_ estimates to account for tracer blood-brain delivery
- Subjects’ age, sex, and genotype to account for individual characteristics associated with TSPO variability
- Tracer dose over subjects’ weight ratio to account for experimental differences

All regressors were normalized to the [0 1] range with min-max normalization, with the minimum and maximum values computed from all the available data. Regions with *low-* and *high- expression* ROIs were adopted for model training with pre-assigned outcomes 0 (i.e. non-inflamed class) and 1 (i.e. inflamed class), respectively.

Specifically, the dataset was divided into a training-validation set (80%, n=56) and a test set (20%, n=16) with a stratified split aiming at maintaining the same proportion of males and females, HABs and MABs, younger (under 30 years old) and older (over 30 years old) individuals between the two groups. Feature selection was performed with the stepwise approach implemented in Matlab (function *stepwiseglm*), with model deviance adopted as a selection criterion. This step was repeated for 100 iterations using a bootstrap resampling approach and only features with a frequency of selection over 80% were kept as relevant features in the model. This approach was adopted to guarantee a more stable and robust selection of model covariates.

The performances of both the full model (with all original regressors) and the reduced model (with only the regressors that passed the stepwise selection) were evaluated on the test set. The ROC curve of method classification - starting from regional **p**_**TSPO**_ estimates - in discriminating tissues with different constitutive TSPO expressions was adopted for the evaluation of their performance.

Once the model set-up was optimized (i.e. the brain regions to adopt for model training and the subset of most informative predictors were selected), the logistic model was trained and tested on [^11^C]PBR28 PET scans on 72, 27, and 26 HCs from three independent datasets (Dataset 1, Dataset 2 and Dataset 3). A random effect was included in the logistic regression model to account for differences in the acquisition between the three datasets, bringing to the definition of a hierarchical logistic regression model ^62,63^. Again, the whole dataset was split into a training and test set with a stratified division accounting for age, sex, genotype, and scanner. After training the model on the training dataset, the model was applied to the test set, and an ROC curve was adopted for the evaluation of model performances.

A leave-one-out approach was finally adopted for the application of the model to each HC. The model was applied for the prediction of the probability **p**_**TSPO**_ for all the ROIs belonging to the cortex and cerebellum (CC ROIs). Hence, the ROIs average **p**_**TSPO**_ across healthy subjects for each dataset was correlated with the measure of TSPO expression provided by mRNA data from the Allen Human Brain Atlas, to assess **p**_**TSPO**_ ability to serve as a proxy of cortical TSPO density in healthy brain. To assess possible dependencies of CC ROIs **p**_**TSPO**_ from the set of covariates of interest, the distribution of CC ROIs **p**_**TSPO**_ was compared between HABs and MABs, as well as between female and male volunteers; similarly, possible correlations between median value across CC ROIs of predicted **p**_**TSPO**_ for each subject and age or dose-over-weight ratio were investigated.

#### Applications to pathological conditions

In order to evaluate the actual feasibility of adopting ROIs **p**_**TSPO**_ as a biomarker of regional neuroinflammatory load in clinical populations, the logistic regression approach was applied to [^11^C]PBR28 data acquired in UHR, SCZ, AD, and FM patients as well as to data acquired with TSPO blocking agent XDB173. A reproducibility analysis was also conducted on test-retest [^11^C]PBR28 data from AD patients. This step aimed to test the ability of the metric **p**_**TSPO**_ to unveil changes in TSPO density and replicate results from previous studies. A summary of the information for each group is reported in **Supplementary Table 1**.

Based on the findings reported in the original references we expected to obtain the following results:

- Increased TSPO expression in total, frontal, and temporal gray matter for UHR and SCZ compared to controls ^41,64^
- Modest widespread cortical increase in TSPO density, with the most pronounced elevation in the frontal and parietal lobes, for FM patients compared to HCs ^43^
- Increased TSPO density in AD patients compared to controls in frontal, parietal, temporal, and occipital regions ^65^; no increase was reported for cerebellum ^66^
- Widespread reduction of tracer-specific binding following XBD173 administration ^44^.

Between-group differences in CC ROIs **p**_**TSPO**_ were analyzed by qualitative comparison of **p**_**TSPO**_ distributions (histograms normalized as relative probability) and by testing of statistical differences between the distributions with a non-parametric test – i.e. the Wilcoxon rank sum test or the Wilcoxon signed-rank test when a paired test was necessary (e.g. for the XBD173 blocking study). For each study, a deviation distribution was also computed as between-groups bin-by-bin difference (case group less control group) of the groups’ relative probability histograms of the CC ROIs **p**_**TSPO**._ Finally, a measure of the magnitude of between-group deviation of CC ROIs p_TSPO_ (Δ_P_) was defined as the percentage sum of the difference distribution for **p**_**TSPO**_ values higher than 0.5. The Δ_P_ provides a measure of increase of the relative probability of high **p**_**TSPO**_ values (higher than 50% probability) for the case group with respect to the control group.

Comparison of CC ROIs **p**_**TSPO**_ distribution was also repeated for each brain lobe (frontal, temporal, parietal, and occipital lobe), as well as for cerebellum ROIs.

In order to test the reproducibility of model estimates, distributions of CC ROIs **p**_**TSPO**_ from test-retest data on AD patients ^42^ were compared, and the intraclass correlation coefficient (ICC) was computed between test and retest **p**_**TSPO**_ estimates for each CC ROI.

### 2.5. Expanding the logistic regression model to [^18^F]DPA-714 and [^11^C]-(*R*)-PK11195 PET imaging

The hierarchical logistic regression model was applied to all 5 datasets, expanding the model to the quantification of [^18^F]DPA-714 and [^11^C]-(*R*)-PK11195 data. The previous model setup, in terms of both TACs subsampling and feature selection, was maintained since the small sample size did not allow for tracer-specific training and optimization of the model. The genotype predictor was set to 1 (corresponding to HAB class) for [^11^C]-(*R*)-PK11195 as the tracer is not susceptible to TSPO polymorphism ^37^. The hierarchical model was adopted for the prediction of CC-ROIs **p**_**TSPO**_ for all HCs (with a leave-one-out approach, excluding the test subject from the training set) and patients (after training the model on all the HCs). The logistic model was thus applied to the cross-sectional analysis - by comparison of ROIs **p**_**TSPO**_ - of 2 studies: the analysis of differences between TREM2 and MCI patients from [^18^F]DPA-714 Dataset 4, and the study of depressed subjects from [^11^C]-(*R*)-PK11195 Dataset 5. Details on compared groups for each study are reported in **Supplementary Table 2**.

Consistently with literature findings, we hypothesized a widespread mild reduction of TSPO signal, as well as a focal reduction in Braak II and Braak III regions, in TREM2 patients compared to MCI ^45^, and a modest increase (given the range of Cohen’s reported in ^46^) of TSPO signal in the insula, prefrontal cortex (PFC), and anterior cingulate cortex (ACC) in depressed subjects compared to HCs. Consequently, distributions of CC ROIs **p**_**TSPO**_ were compared between the MCI and TREM2 groups, and ad hoc analyses of differences in **p**_**TSPO**_ for Braak II and Braak III regions were performed. Concerning the depression study, **p**_**TSPO**_ of insula, PFC, and ACC was compared between the depressed and HC groups.

### 2.6. Application to a rat model of LPS-induced neuroinflammation

Data included a total of 20 dynamic [^18^F]DPA-714 brain PET scans on rats gathered from a previous study of LPS-induced neuroinflammation^18^. The first dataset included 4 PET scans carried out 4 days after intracerebral (ic) administration of LPS from Escherichia coli 0111: B4 (Sigma) (*ic-LPS*) into the right dorsal striatum. The second dataset included 8 PET scans carried out around 24 hours after peripheral administration of LPS with intraperitoneal (ip) injection (*ip-LPS*) and 8 control scans performed 24 hours after ip vehicle injection (*Vehicle*). More details on experimental protocols, data acquisition, and image analyses can be found in the original reference ^18^. Overall, the data analysis focused on 13 ROIs TACs extracted for each animal using the 3D rat brain atlas template as employed by the VivoQuant 2.0 (Invicro LLC) software.

Previous evidence has shown that the *ic-LPS* striatal injection challenge induces a robust focal inflammatory lesion in the ipsilateral hemisphere, while the contralateral side shows no inflammatory reaction ^18,67^. Model training was thus performed on *ic-LPS* scans. A subset of the 13 ROIs in the ipsilateral side of the lesion and the respective ROIs in the contralateral (non-lesioned) side were assumed as *high- and low-expression ROIs*, respectively. Specifically, TACs AUC was compared between the two hemispheres for each brain ROI with a paired t-test, and ROIs showing a statistically significant difference in the TAC AUC after false discovery rate (FDR) correction for multiple comparisons were selected for model training. Starting from the logistic regression model setup and features selection adopted in human studies, the model was further simplified by the exclusion of age and sex (which were standardized in the group of animals) but also genotype from model predictors. After model training, given the small sample size of the *ic-LPS* training set, the significance of predictors was tested by application of the Wald test.

The model was applied for the computation of brain ROIs **p**_**TSPO**_ for *ip-LPS* and *Vehicle* scans. Then, regional **p**_**TSPO**_ estimates were compared between the two groups for each ROI by application of the Wilcoxon rank-sum test and FDR correction for multiple comparisons.

## 3. Results

### 3.1. Logistic regression model for [^11^C]PBR28 PET quantification

#### Model training

**Figure 2.**
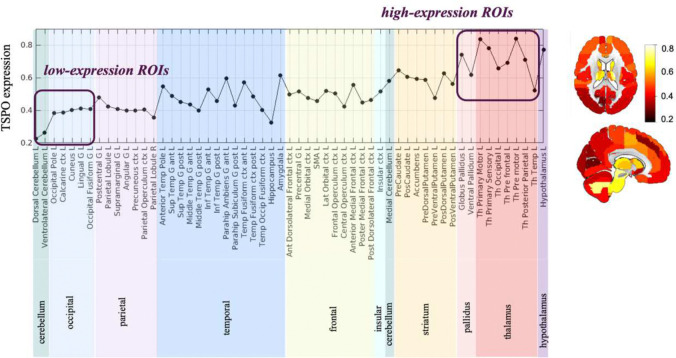
TSPO gene expression map The figure shows regional TSPO gene expression derived from the Allen Human Brain Atlas for anatomical ROIs defined by the CIC atlas. A graph showing regional values of gene expression in the left hemisphere is shown on the left. Representative slices of the map of TSPO expression are represented on the right. *Low-* and *high-expression ROIs* are highlighted in the graph.

Analysis of the topological pattern of TSPO gene expression (Figure 2), derived from the data of the Allen Human Brain Atlas, reveals high TSPO expression in subcortical regions, particularly in the pallidum and thalamic regions. Brain cortex ROIs exhibit lower but highly variable levels of gene expression. The cerebellum shows low, though not negligible, TSPO expression, except in the medial regions. Following this pattern, the occipital lobe, along with the dorsal and ventrolateral regions of the cerebellum, were selected as *low-expression ROIs*, while the thalamic and pallidus regions as *high-expression ROIs*. These regions, which are expected to manifest, in physiological conditions, respectively low and high density of TSPO, were used to train the logistic model.

A preliminary step of feature selection was performed with an iterative stepwise approach. Starting from the initial selection of model predictors (i.e. ROIs TACs samples at 1.25, 4.50, 13.50, 30, 50, and 75 minutes as well as ROIs *K*_*1*_ and subjects’ age, sex, genotype, and tracer dose over subjects’ weight ratio), the resulting set of selected features included ROIs TAC samples at 1.25, 13.5 and 50 minutes, together with age, genotype, and the *K*_*1*_ microparameter. Notably, sex was excluded from model predictors (resulting in a non-significant predictor). ROC curves reported in **Supplementary Figure 1a**, show good classification performances of TSPO tissue expression, in terms of sensitivity and specificity, of the logistic regression model on Dataset 1. Comparable levels of sensitivity and specificity are reported for the full model (AUC=0.92, sensitivity=0.87, specificity=0.84) - including all the original predictors –and the reduced model (AUC=0.92, sensitivity=0.89, specificity=0.86) – with only the subset of predictors identified with the features selection. Similarly, good performances of classification are reported from the application of the hierarchical logistic regression model, which included an additional random effect to model differences between datasets, on HCs [^11^C]PBR28 scans from Dataset 1, 2, and 3, as shown by the ROC curves in **Supplementary Figure 1b**. Again, similar values of specificity and sensitivity are reported for the full (AUC=0.93, sensitivity=0.83, specificity=0.87) and reduced (AUC=0.92, sensitivity=0.85, specificity=0.89) models, confirming the reliability of adopting the subset of selected predictors.

#### Validation in healthy controls

**Figure 3.**
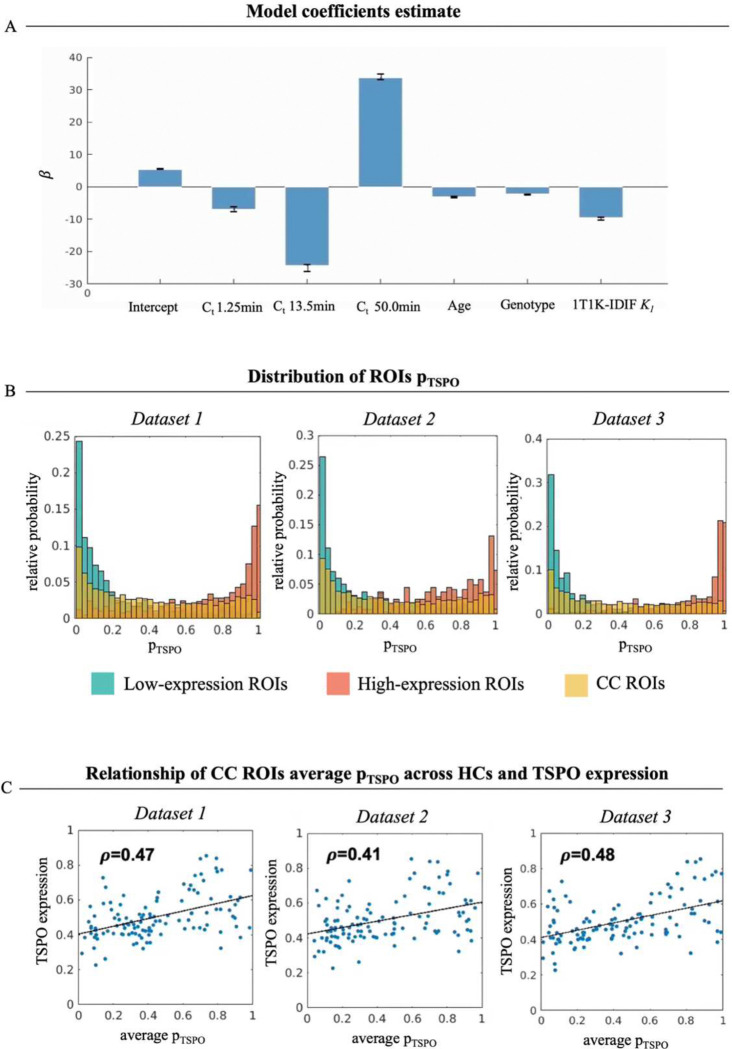
Leave-one-out training and application to [^11^C]PBR28 healthy control scans Panel A reports the results of the coefficient estimates for each predictor: colored bars represent the values of *β* estimates obtained when training on the whole HC cohort, while error bars represent the variability of estimates between the different iterations of the leave-one-out approach; panel B shows the relative probability of *low-expression* (green), *high-expression* (orange) and CC (yellow) ROIs p_TSPO_ for each of the three datasets; panel C represents correlation between HCs’ average CC ROIs p_TSPO_ and the regional TSPO gene expression [HC=healthy control].

To evaluate the ability of the logistic regression approach to provide a physiological measure of TSPO density, firstly the model was applied to the quantification of [^11^C]PBR28 scans on HCs employing a leave-one-out approach for model training and prediction. Results of leave-one-out training show low variability among the various iterations in model coefficient estimates (coefficient of variation cv=1.5±0.4%; mean±std across model predictors), as represented in Figure 3a. Coefficients related to ROI TAC samples are the highest in absolute value, particularly for samples at 13.5 minutes (tstat=−20.9) and 50 minutes (tstat=24.9). However, while model coefficient shows a positive sign for the TACs sample at 50 min, an inverse relationship is reported for the early samples of tissue kinetics. Lower but non-negligible contribution is given by age (tstat=−9.5), genotype (tstat=−15.31), and *K*_*1*_ predictors (tstat=−13.35).

As expected, in a healthy condition the predicted **p**_**TSPO**_ shows values close to zero and 1 respectively for the *low-* and *high-expression ROIs*, as clearly visible from histograms of **p**_**TSPO**_ for the HC cohorts of the three different [^11^C]PBR28 datasets (Dataset 1, 2 and 3) reported in Figure 3b. Histograms of CC ROIs **p**_**TSPO**_ on the other hand show high variability in the **p**_**TSPO**_ values. This variability partially reflects the topological pattern of TSPO gene expression in the brain, as shown by the high correlation between the across-subjects average of CC ROIs **p**_**TSPO**_ and the pattern of gene expression from the Allen Human Brain Atlas for each of the three HC cohorts (Dataset1: ρ=0.47; Dataset2: ρ=0.41; Dataset3: ρ=0.48, all p<0.05; Figure 3c).

Consistent with the set of covariates included in the model, post hoc analysis reveals no statistical differences in **p**_**TSPO**_ when comparing results for HABs and MABs, and no significant correlation with either age or dose-over-weight ratio. However, statistically significant differences in **p**_**TSPO**_ distributions are reported when comparing males to females, with females showing lower **p**_**TSPO**_ values (**Supplementary Figure 2**).

#### Validation in clinical populations

**Figure 4.**
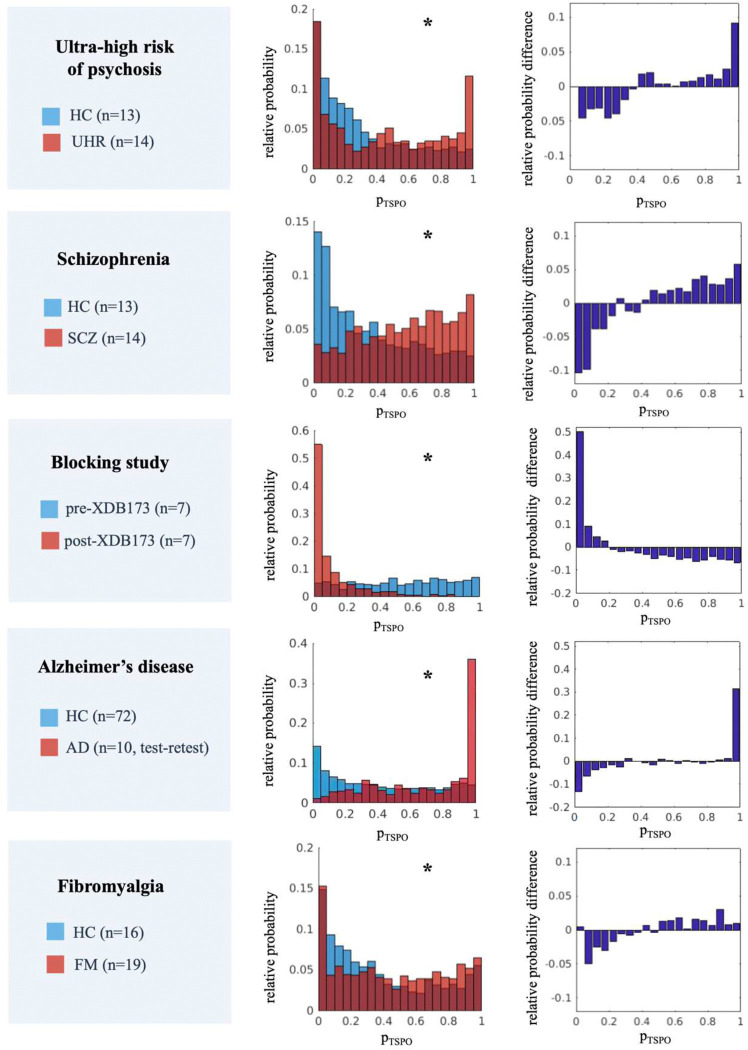
Cross-sectional analysis – Comparison of CC ROIs p_TSPO_ relative distribution Each panel shows, for each study under investigation, the comparison of the group relative probability of CC ROIs **p**_**TSPO**_ (on the left) and the difference between the two relative probabilities (on the right) [HC=healthy control; UHR=ultra-high risk of psychosis; SCZ=schizophrenia; AD=Alzheimer’s disease; FM=fibromyalgia; * Wilcoxon (paired or unpaired) test pvalue<0.0001].

Consistent with the study hypothesis, the comparison of group distributions for CC ROIs **p**_**TSPO**_ (Figure 4) shows statistically significant increases in UHR, SCZ, AD, and FM patients with respect to HCs. In contrast, cortical and cerebellar **p**_**TSPO**_ demonstrates a significant reduction after target blocking via XBD173 administration. The magnitude of between-group deviations Δ_*P*_ revealed the highest deviation in the case of XBD173 blocking (Δ_*P*_=−52%), followed by AD (Δ_*P*_=+32%), SCZ (Δ_*P*_=+29%), and UHR (Δ_*P*_=+18%) patients; a mild effect was reported for FM patients (Δ_*P*_=+13%).

Between-group comparisons of CC ROIs **p**_**TSPO**_ for each anatomical lobe show a significant increase for the UHR, SCZ, and AD with respect to HCs and a significant decrease in the case of XDB173 blocking for all four lobes (**Supplementary Figure 3**). FM patients show a significant increase only in the frontal, occipital, and parietal lobes. In all studies, significant differences were also found when comparing cerebellum **p**_**TSPO**_ between the two groups. The analysis of test-retest data on AD patients showed high reproducibility both in terms of the similarity of CC ROIs **p**_**TSPO**_ histograms (Wilcoxon rank sum test p-value>0.05, Δ_*P*_=−2%) and ICC, with 74% ROIs having ICC ≥ 0.7 (**Supplementary Figure 4**).

### 3.2. Expanding the hierarchical model to [^18^F]DPA-714 and [^11^C]-(*R*)-PK11195

**Figure 5.**
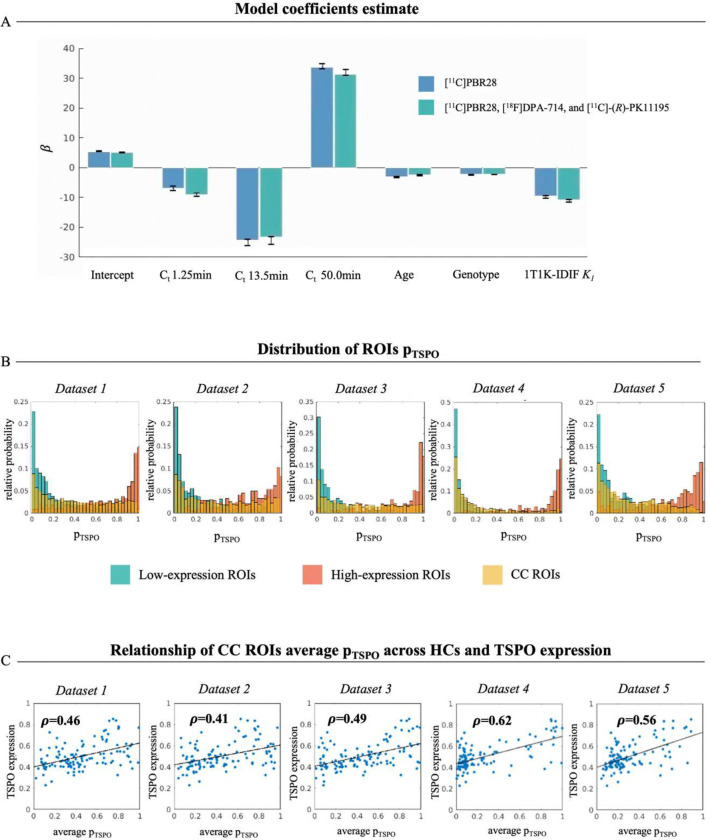
Leave-one-out training and application to [^11^C]PBR28, [^18^F]DPA-714 and [^11^C]-PK11195 healthy control scans Panel A reports the results of the coefficient estimates for model training on [^11^C]PBR28, [^18^F]DPA-714, and [^11^C]-(*R*)-PK11195 HCs (green), compared to results of training on only [^11^C]PBR28 data (blue): colored bars represent the values of *β* estimates obtained when training on the whole HC cohort, while error bars represent the variability of estimates between the different iterations of the leave-one-out approach; panel B shows the relative probability of *low-expression* (green), *high-expression* (orange) and CC (yellow) ROIs **p**_**TSPO**_ for each of the three datasets; panel C represents correlation between the across HCs average of CC ROIs **p**_**TSPO**_ and the regional TSPO gene expression [HC=healthy control].

Re-training without feature optimization of the hierarchical model with the inclusion of HC cohorts from [^18^F]DPA-714 and [^11^C]-(*R*)-PK11195 datasets gave consistent results to the [^11^C]PBR28 model training (Figure 5). Coefficient estimates show a comparable pattern to the one obtained from training on [^11^C]PBR28 HCs in terms of coefficients sign, absolute value, and variability among leave-one-out estimates. As for the [^11^C]PBR28 model, histograms of predicted ROIs **p**_**TSPO**_ in HCs of each of the five datasets show values respectively close to zero and 1 for the *low-* and *high-expression ROIs*, and relatively low but highly variable values for CC ROIs **p**_**TSPO**_. Across-subjects average of CC ROIs **p**_**TSPO**_ shows a good correlation to the regional level of TSPO gene expression (Dataset 1: ρ=0.46; Dataset 2: ρ=0.41; Dataset 3: ρ=0.49; Dataset 2: ρ=0.62; Dataset 3: ρ=0.56).

Results of model application to cross-sectional analysis on [^18^F]DPA-714 (Dataset 4) show a significant but modest increase of CC ROIs **p**_**TSPO**_ for the TREM2 with respect to the MCI group (Wilcoxon rank sum test p-value<10^−5^, Δ_*P*_=+6%) and no statistical difference when comparing Braak2 (Cohen’s d = −0.134) and Braak3 regions (Cohen’s d = 0.392), differently from original publication. Cross-sectional analysis of [^11^C]-(*R*)-PK11195 scans showed a modest increase of **p**_**TSPO**_ of insula (Cohen’s d = 0.138), PFC (Cohen’s d = 0.081), and ACC (Cohen’s d = 0.163) for depressed subjects compared to HCs but none of this region reaches statistical significance due to the high intersubject variability in **p**_**TSPO**_ (insula: sd_HC_=0.234, sd_MD_=0.201; PFC: sd_HC_=0.253, sd_MD_=0.202; ACC: sd_HC_= 0.265, sd_MD_= 0.205).

### 3.3. Application to a rat model of LPS-induced neuroinflammation

**Figure 6.**
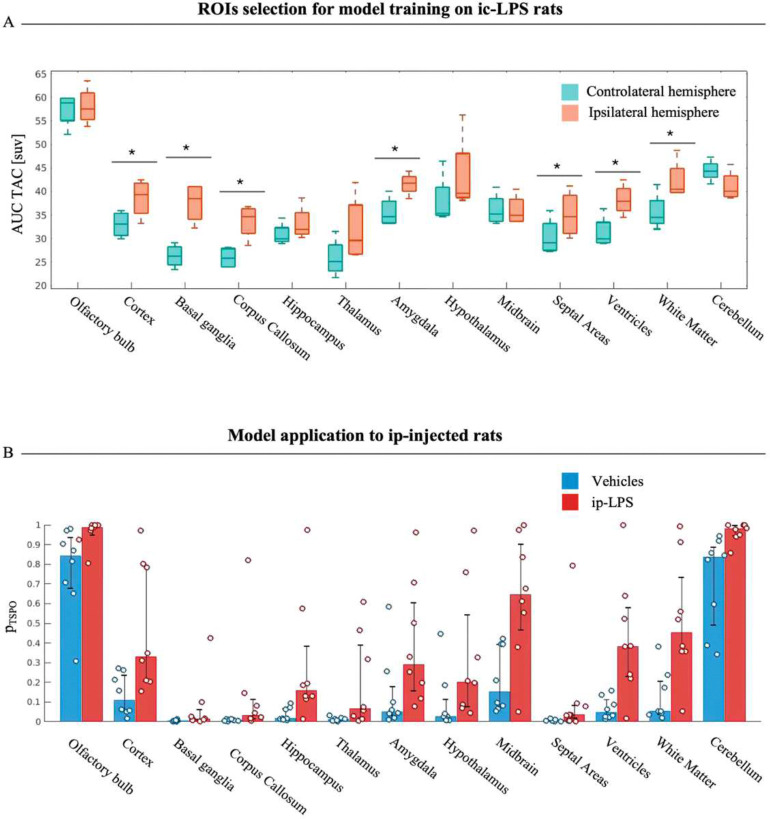
Application to a rat model of LPS-induced neuroinflammation Panel A shows the comparison of the area under the curve of TACs in SUV of homologous ROIs in *ic-LPS* rats; a significant difference is reported for cortex, basal ganglia, corpus callosum, amygdala, septal area, ventricles and white matter. Panel B shows the comparison of regional **p**_**TSPO**_ between the *Vehicle* and *ip-LPS* groups.

ROIs TACs AUC showed significant difference between the ipsilateral and contralateral hemisphere for cortex, basal ganglia, corpus callosum, amygdala, septal area, ventricles, and white matter (Figure 6a). These regions were thus employed for model training. Only the predictor corresponding to the TAC sample at 13.5 min resulted in statistical significance from the Wald test (z-score>1.96). The model was thus trained with the inclusion of only this predictor. Model application to LPS and vehicle ip-administered rat scans showed significant increase in **p**_**TSPO**_ values in the *ip-LPS* with respect to the *Vehicle* group (Figure 6b).

## 4. Discussion

We developed a novel TSPO PET analytical framework that exploits the raw PET signal to provide a statistical measure of brain TSPO density. We tested it on multiple TSPO radiotracers, scanners, and imaging facilities, replicating literature findings in different (even if not all) clinical cohorts. The method does not utilize blood-based kinetic modeling and requires dynamic scanning of a maximum of 60 minutes from tracer injection, reducing experimental time.

### Proposed improvements

The proposed analytical framework could potentially address the following limitations of standard TSPO PET quantification:

1. In contrast to standard kinetic modeling, the method does not require any arterial blood samples, nor laborious procedures for the quantification of plasma tracer activity and the correction of metabolite radioactivity. The use of the *1T1K-IDIF* method ^48^ allowed for a non-invasive estimation with an IDIF of the *K*_*1*_ microparameter.
2. The approach does not require the identification of a reference region, which is particularly challenging for TSPO PET imaging, and, as it avoids any normalization for the reference TAC, helps in the identification of mild global effects related to a disorder. Even when more sophisticated data-driven approaches like supervised clustering are adopted for the identification of the reference region, the identified region is often still contaminated at some level by specific binding of the tracer.
3. The inclusion in the model of the *K*_*1*_ microparameter - despite limitations of the *1T1K-IDIF* approach adopted for the estimation ^48^ - allows taking into account possible alterations of the tracer delivery rate linked to modulation of cerebral blood flow or BBB permeability in pathological conditions.
4. The adoption of a regression approach, with genotype and age included as model predictors, allows us to explain part of the inter-individual variability in the TSPO PET signal in both physiological and pathological conditions that limits the tracer statistical power in assessing the presence and progression of neuroinflammation in clinical applications.
5. The definition of a hierarchical model allows the modeling of possible batch effects linked to differences in scanners and protocols adopted for data acquisition.

The proposed methodology has the potential to serve as a standardized tool for neuroinflammation assessment, facilitating its integration into clinical research and potentially into routine clinical practice for more personalized and accurate diagnosis and/or monitoring of neuroinflammatory conditions.

### Highlights from HC analysis

The validation of the logistic regression model (both hierarchical and not) shows excellent performances of classification of *low-* and *high-expression ROIs* in HCs in terms of specificity and sensitivity of the model. The preliminary interpolation of the ROI TACs on a less dense time grid allows for a reduction of the number of model predictors but also of possible problems linked to collinearity between variables. The stepwise features selection analysis allows for further reduction in the number of variables and possible overfitting issues.

The application of the approach to the quantification of [^11^C]PBR28, [^18^F]-DPA714, and [^11^C]-(*R*)-PK11195 on healthy control scans showed a good concordance between the pattern of TSPO expression predicted by the model and the topology of gene expression defined by mRNA data from the Allen Human Brain Atlas. Previous studies have already investigated the concordance between PET imaging and genetic data on regional TSPO expression with inconsistent results ^68,69^. This new quantification approach can map constitutive TSPO density better than any previous quantification method. The model demonstrated the ability to replicate the expected topological pattern of TSPO in healthy individuals. The training of the hierarchical model on [^11^C]PBR28 data, as well as the retraining of the hierarchical model with the inclusion of [^18^F]DPA-714 and [^11^C]-(*R*)-PK11195 HC scans, gave consistent results, both in terms of CC ROIs **p**_**TSPO**_ distributions and model coefficient estimates.

### Model Covariates

Although sex was not retained as a significant predictor in the features selection step, post hoc analyses showed significantly lower values of CC ROIs **p**_**TSPO**_ for female than male HCs, thus highlighting sex as a possible factor affecting the level of TSPO in the brain. These results are in line with evidence from the literature reporting a dependency of TSPO density on sex, despite mixed results being shown so far, demonstrating increased or decreased TSPO expression in female compared to male individuals according to the type of quantification and metrics adopted ^38–40^. Given the dependence of **p**_**TSPO**_ on sex, significant between-group differences in the proportion of males and females were thus checked for each case study under investigation. The chi-squared test found no significant differences between each pair of groups under study.

On the other hand, a posteriori analysis of **p**_**TSPO**_ with age and genotype included as model predictors did not show any association, highlighting that these effects were effectively removed by the model. Interestingly, the model coefficient for *K*_*1*_ shows an inverse association between tracer blood-to-brain exchange *K*_*1*_ and the **p**_**TSPO,**_ indicating that lower tracer delivery values are associated with a higher inflammatory load. This is consistent with the theory linking peripheral with central inflammation proposed by Turkheimer et al ^36^.

### Cross-sectional analysis on [^11^C]PBR28 data

The application of the method to cross-sectional analyses on [^11^C]PBR28 data gave promising results, replicating evidence from previous studies for different cohorts and showing good reproducibility of ROIs **p**_**TSPO**_ estimates. As expected from previous evidence ^41^, a widespread increase in TSPO density was reported in the whole cortex when comparing ultra-high risk of psychosis and schizophrenia patients to healthy controls. Similarly, a reduction in the whole brain TSPO PET signal was shown after XDB173 target blocking ^44^. An increase in **p**_**TSPO**_ reflecting an increase in TSPO density for frontal, parietal, temporal, and occipital lobes was reported for Alzheimer’s disease patients in line with previous general references ^66^. Regarding fibromyalgia patients, a widespread gray matter **p**_**TSPO**_ increase as well as an increase for frontal and parietal lobes were shown, in line with the results from the original reference ^43^.

### TSPO expression in the cerebellum

It is worth mentioning that, in all the [^11^C]PBR28 cross-sectional studies performed, statistically significant differences between groups in cerebellum ROIs **p**_**TSPO**_ were reported. Given the ubiquitous widespread expression of TSPO in the brain, no anatomical region has been demonstrated to completely lack any specific binding to the target. The cerebellum has often been adopted as a pseudo-reference region for the computation of relative measures of TSPO load and has been validated for use in Alzheimer’s disease studies ^70^. However, given the high cellular heterogeneity and TSPO displacement, the cerebellum may not represent an ideal reference region for TSPO PET imaging studies, and its adoption should be carefully checked for each brain condition ^44^. The cerebellum is also close to the confluences of sinuses, where increases in TSPO PET signal, possibly from cerebrospinal fluid coming out of the inflamed brain, have been found to be associated with both central and peripheral inflammation ^34^.

### Extension to other tracers

While the logistic model successfully detected TSPO alterations in cross-sectional studies with [^11^C]PBR28 data, we were not able to fully replicate results for [^18^F]DPA-714 and [^11^C]-(*R*)-PK11195 datasets. This discrepancy could be due to several factors. First, the sample size was relatively small, with only 8 [^18^F]DPA-714 scans for the TREM2 carriers and mild cognitive impaired subject groups, and only 25 healthy controls for [^11^C]-(*R*)-PK11195 data. Second, the model’s settings – ranging from the time grid adopted for TACs subsampling to the selection of features – were optimized specifically for [^11^C]PBR28 and could be suboptimal for the quantification of [^18^F]DPA-714 and [^11^C]-(*R*)-PK11195. Differences between tracers extend beyond variations in affinity to the specific pharmacokinetics of each tracer as well as its dependencies on genetic polymorphism, thus opening the necessity for the development of tracer-specific optimization and training of the model. Consequently, the possibility of adopting the model for the quantification of TSPO density from [^18^F]DPA-714 and [^11^C]-(*R*)-PK11195 should be further explored. Larger cohorts of HCs and patients will be necessary for model optimization and training on [^18^F]DPA-714 and [^11^C]-(*R*)-PK11195 data. Finally, it is worth mentioning that original studies for both the TREM2 and the depression cohorts reported very mild alterations in TSPO density; testing the method on new cohorts with evidence of stronger effects of the disease will help to clarify the actual feasibility of expanding the method to other TSPO tracers.

### Model application to the study of LPS-induced brain inflammation in rats

A slightly different approach was adopted for the application of the logistic regression method to rat models of neuroinflammation in order to account for differences between species and adapt the model to the peculiar experimental protocol. Regional TACs derived from ic-LPS scans from ipsilateral (lesioned) and contralateral (non-lesioned) hemispheres - with respect to LPS ic-injection – provide examples of [^18^F]DPA-714 kinetics from inflamed and not-inflamed regions respectively and were thus adopted for model training. Despite the small sample size of the ic-LPS dataset, this choice guaranteed a more controlled setting for the training of the logistic regression model as the reference classes for TSPO constitutive expression (class 0) and overexpression (class 1) were histologically validated. Moreover, given the standardized characteristics of rats, sex and age were not included as model predictors, simplifying the model construction and, therefore, its parameter identification.

Despite the simplifications and the small sample size of the training set, the logistic regression model was able to replicate the regional pattern of TSPO expression in vehicle scans reported in the original reference ^18^. Additionally, the model allowed us to unveil the expected brain widespread increased inflammatory load 24h after the intraperitoneal LPS challenge, corroborating the original results.

### Limitations and future directions

The use of pre-existing data, which were collected with the use of different scanners and specific acquisition protocols, represents a limitation of this study. Image characteristics in terms of spatial and temporal resolution and signal-to-noise ratio would particularly affect the IDIF extraction and 1T1K-IDIF regional *K*_*1*_ estimation. However, the use of a hierarchical model allowed us to partially account for possible differences linked to discrepancies in data collection. Moreover, cross-sectional analyses were always conducted between scans sharing the same scanner and acquisition protocol. The only exception was represented by the shorter acquisition length of [^11^C]PBR28 AD scans, with only 60 minutes against the 90 minutes of acquisition of the HC group, despite general analogies between the acquisition protocols and the use of the same scanner for the data collection. Since the final configuration of the designed model included only TAC samples corresponding to the mid-frame time of 1.25, 13.5, and 50 minutes, we were still able to apply the logistic regression method to the AD cohort.

Another general limitation of the study is related to model assumptions. According to its formulation, the model assumes a consistent effect of the covariates for all regions. This assumption is reasonable given that the approach is designed for the only application to cortical regions of the brain and cerebellum. However, more complex formulations of the methodological framework allowing for a regional modulation of model coefficients should be explored. Similarly, different formulations accounting for possible non-linear relationships and interactions between covariates should be tested. The model could also be improved with the inclusion of further covariates such as BMI, stress, and the presence of comorbidities, which were not available for the data under study but could represent significant factors affecting brain TSPO load. Moreover, additional data and analyses will be fundamental to test the possibility of extending the methodological framework from ROI- to voxel-wise quantification and derive parametric maps of TSPO density. Furthermore, the analysis of subject-specific distributions of **p**_**TSPO**_ metrics could allow for the derivation of individual scores of neuroinflammation, but this would require additional investigation.

Finally, it must be highlighted that the Allen Human Brain Atlas gene expression data have been derived from only six postmortem adult brains, with data related to the right hemisphere only available for two donors. This highlights the intrinsic limitation of this data in the investigation of gene expression in the human brain.

### Conclusions

We developed a new blood- and reference-free analytical framework for the quantification of regional brain density. Model validation supports the use of the **p**_**TSPO**_ metrics in the study of neuroinflammation and the application of this approach to data collected with different scanners and acquisition protocols. This method could be applied to the quantification of different TSPO tracers, given adequate reference sample size for a tracer-specific model optimization and parameter training. Further studies and larger sample sizes are necessary for the optimization of the methodology and the derivation of subject-specific scores of neuroinflammation.

## Figures and Tables

**Table 1:** Demographic and acquisition information for the 5 datasets under study

	Dataset 1	Dataset 2	Dataset 3	Dataset 4	Dataset 5
*Tracer*	[^11^C]PBR28	[^11^C]PBR28	[^11^C]PBR28	[^18^F]DPA-714	[^11^C]-(*R*)-PK11195
*Affiliation*	KCL	MGH	MGH	KCL	KCL
*Scanner type*	Siemens Biograph^™^ TruePoint^™^ PET·CT scanner	dedicated brain PET scanner within the bore of a Siemens 3T Tim Trio MRI scanner	Siemens Biograph mMR whole-body PET/MR scanner	SIEMENS Biograph mMR PET/MRI	GE SIGNA PET/MR
*Age (m±std)*	38 ± 19	47 ± 13	55 ± 15	39 ± 19	37 ± 8
*Sex (#M,#F)*	84, 34	15, 31	12, 14	31, 26	26, 50
*Genotype (#HAB,#MAB)*	87, 31	30 , 16	13 , 13	37 , 20	/
*Dose (mean±std)*	328.10 ± 32.79	488.16 ± 54.99	519.44 ± 63.04	186.88 ± 10.00	365.53 ± 50.89
*Clinical populations*	HC, UHR, SCZ, XDB173 blocking, AD	HC, FM	HC	HC, TREM2, MCI	HC, MD

HC=healthy control; UHR=ultra-high risk of psychosis; SCZ=schizophrenia; AD=Alzheimer’s disease; FM=fibromyalgia; TREM2=TREM2 p.R47H carriers; MCI=mild cognitive impairment; MD=mild depressive disorders; m=mean; std=standard deviation; HAB=high affinity binding; MAB=mixed affinity binding; M=males; F=females.
